# Non-neuronal cardiac acetylcholine system playing indispensable roles in cardiac homeostasis confers resiliency to the heart

**DOI:** 10.1186/s12576-020-00787-6

**Published:** 2021-01-18

**Authors:** Shino Oikawa, Yuko Kai, Asuka Mano, Hisayuki Ohata, Atsushi Kurabayashi, Masayuki Tsuda, Yoshihiko Kakinuma

**Affiliations:** 1 Department of Bioregulatory Science (Physiology), Nippon Medical School, Graduate School of Medicine, Sendagi, Bunkyo-ku, Tokyo, 113-8602 Japan; 2 Department of Pathology, Kochi Medical School, Nankoku, Kochi, 783-8505 Japan; 3 Institute for Laboratory Animal Research, Kochi Medical School, Nankoku, Kochi, 783-8505 Japan

**Keywords:** Cardiac glucose metabolism, Nitric oxide, The vagus nerve, Reactive oxygen species, The non-neuronal cholinergic system

## Abstract

**Background:**

We previously established that the non-neuronal cardiac cholinergic system (NNCCS) is equipped with cardiomyocytes synthesizes acetylcholine (ACh), which is an indispensable endogenous system, sustaining cardiac homeostasis and regulating an inflammatory status, by transgenic mice overexpressing choline acetyltransferase (ChAT) gene in the heart. However, whole body biological significances of NNCCS remain to be fully elucidated.

**Methods and results:**

To consolidate the features, we developed heart-specific ChAT knockdown (ChATKD) mice using 3 ChAT-specific siRNAs. The mice developed cardiac dysfunction. Factors causing it included the downregulation of cardiac glucose metabolism along with decreased signal transduction of Akt/HIF-1alpha/GLUT4, leading to poor glucose utilization, impairment of glycolytic metabolites entering the tricarboxylic (TCA) cycle, the upregulation of reactive oxygen species (ROS) production with an attenuated scavenging potency, and the downregulated nitric oxide (NO) production via NOS1. ChATKD mice revealed a decreased vagus nerve activity, accelerated aggression, more accentuated blood basal corticosterone levels with depression-like phenotypes, several features of which were accompanied by cardiac dysfunction.

**Conclusion:**

The NNCCS plays a crucial role in cardiac homeostasis by regulating the glucose metabolism, ROS synthesis, NO levels, and the cardiac vagus nerve activity. Thus, the NNCCS is suggested a fundamentally crucial system of the heart.

## Introduction

We previously reported that the heart possesses a critical system to support the fundamental cardiac functions using components equipped with a cardiomyocyte to synthesize acetylcholine (ACh). This system has been called the non-neuronal cardiac cholinergic system (NNCCS) or non-neuronal ACh (NNA) in the heart [1]. Thus far, many research groups have globally studied and reported that the NNCCS or NNA possesses decisive cardioprotective roles, e.g., hypertrophy suppression, inhibition of oxygen consumption and reactive oxygen species (ROS) production, and sustaining gap junction function in physiological and pathological conditions [2–8]. Our previous study clearly demonstrated that the NNCCS augmentation, represented by a transgenic mouse overexpressing cardiomyocyte-specific choline acetyltransferase gene (ChAT tg), conferred accelerated ischemia and hypoxia-resistant potency on the heart [9]. Then, it has been evidenced that the NNCCS plays crucial roles in cardiac homeostasis, for examples, cell–cell communication [7], angiogenesis [9, 10], oxygen consumption [1, 7], and anti-ischemia and hypoxia potency [7, 9].

In contrast, a few studies investigating biological significances of ChAT gene deletion on mice revealed distinct phenotypes [11, 12], including lethality after birth or nothing specifically in ordinary conditions, although the conditional knockout mice of vesicular ACh transporter (VAChT) and ChAT genes supported the concept that NNCCS is critical for cardiac homeostasis in common [6, 11, 12]. Therefore, further studies should be needed whether loss of ChAT gene can influence the cardiac and extra-cardiac functions or not.

To perform that, we developed mice with heart-specific characteristic siRNAs overexpression, which recognized ChAT mRNA specific 3′-UTR elements (ChATKD). They showed several cardiac and extra-cardiac phenotypes, remarkably in contrast to those of ChAT tg. These ChATKD phenotypes again supported the findings from the previous studies [12], and further indicated that the NNCCS plays a crucial role not only in the heart but also in the brain as its intrinsic self-defense mechanism.

## Methods

### Development of transgenic mice

#### ChATKD mice

To construct a transgene vector with a murine α-myosin heavy chain promoter (α-MHC), we used the pcDNA^TM^6.2-GW/EmGFP-miR vector (BLOCK-iT™ Pol II miR RNAi Expression Vector Kit with EmGFP), in which three ChAT mRNA 3′-UTR-specific siRNAs, which were commercially optimized and complementary to a specifically determined sequence of the 3′-UTR, were annealed and tandemly included together in the specific vector region (ThermoFisher Scientific K.K. Invitrogen, Tokyo, Japan), as previously reported in our study, where the RNAi expression system was confirmed to work [7]. After the siRNA insertion, the vector region, including siRNAs, internal ribosome entry site IRES, and EmGFP, was transferred into a transgene cassette vector equipped with a murine α-myosin heavy chain promoter. The promoter was previously used for developing the heart-specific ChAT overexpressing tg mice [9], where it was confirmed that the transgene protein expression was predominantly upregulated in the ventricles. The three ChAT-specific siRNAs were commercially available from Invitrogen™. Additionally, they were validated by the Invitrogen™ to specifically downregulate ChAT mRNA expression, as follows:

a) TGCTGATAACAGGCTCCATACCCATTGTTTTGGCCACTGACTGACAATGGGTAGAGCCTGTTAT, and CCTGATAACAGGCTCTACCCATTGTCAGTCAGTGGCCAAAACAATGGGTATGGAGCCTGTTATC;

b) TGCTGATCACAGCAGGGCTAGAGTTGGTTTTGGCCACTGACTGACCAACT CTACCTGCTGTGAT, and CCTGATCACAGCAGGTAGAGTTGGTCAGTCAGTGGCCAAAACCAACTCTAGCCCTGCTGTGATC,

c) TGCTGTGCAGAAGGTGATGGCCTCAGGTTTTGGCCACTGACTGACCTGA GGCCCACCTTCTGCA, and CCTGTGCAGAAGGTGGGCCTCAGGTCAGTCAG TGGCCAAAACCTGAGGCCATCACCTTCTGCAC.

After obtaining the final vector construct which includes 3 kinds of ChAT siRNAs, the linear part of the transgene was prepared by restriction enzymes and used to obtain the cardiac specific ChAT siRNAs overexpressing (ChATKD) mice.

**Fig. 1:**
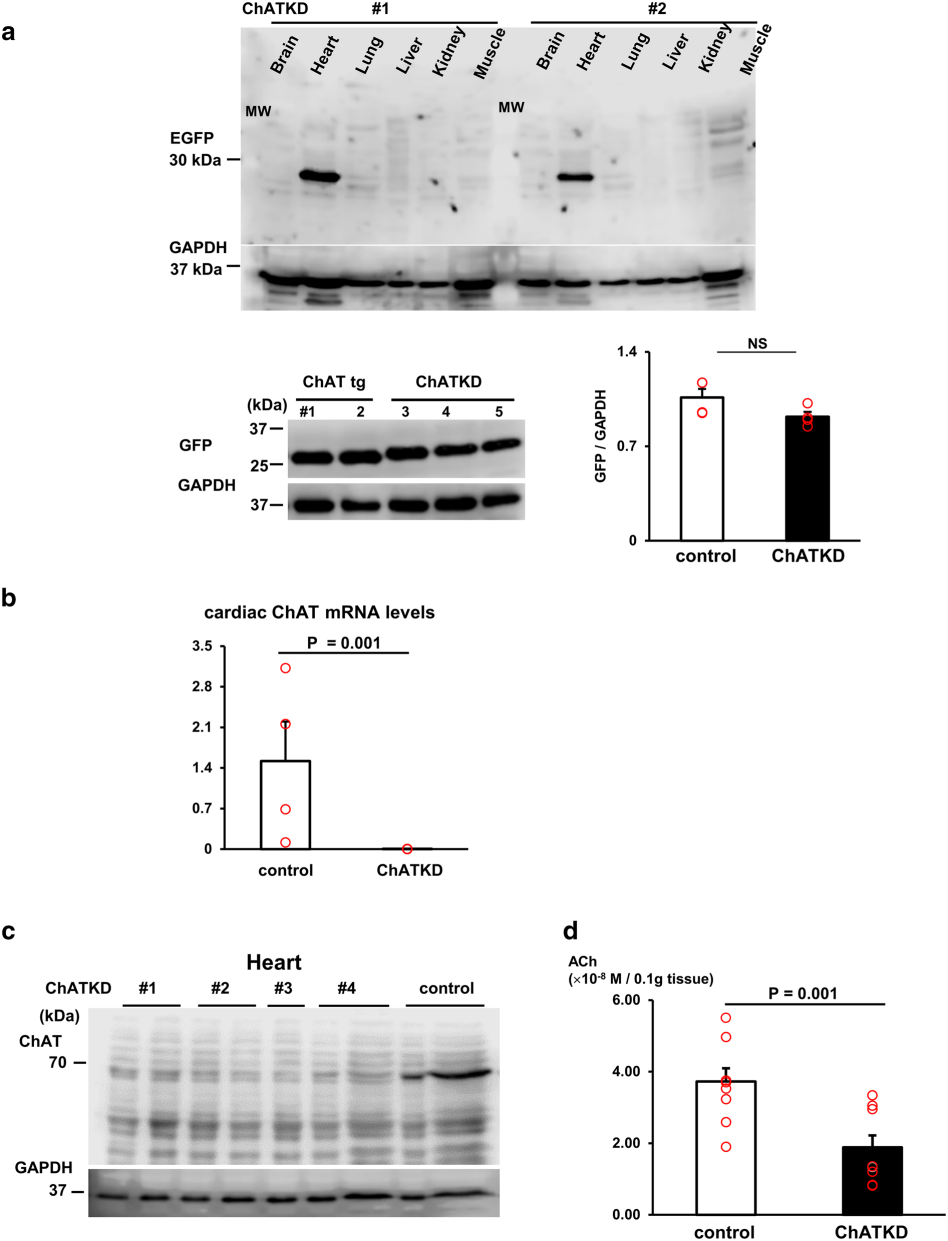
Characterization of the choline acetyltransferase knockdown (ChATKD) transgene in the heart. **a** Knockdown transgene of ChAT-specific sequences for miRNA synthesis is verified to be restrictively expressed in the heart by the GFP protein expression. GFP expression levels in ChATKD mice hearts are comparable to those in ChAT tg mice hearts. **b** ChAT mRNA expression is lower in the ChATKD heart than in control mice (*P* = 0.001). **c** Cardiac protein expression levels of ChAT are lower in the ChATKD heart than in the control heart. **d** An ACh content is lower in the ChATKD heart than in the control heart (*P* = 0.001)

We developed 3 lines of ChAT KD mice overexpressing simultaneously three siRNAs, which possessed the same specific phenotypes as revealed in this study. In contrast, mice overexpressing only one ChAT siRNA, not three, were used as controls because they failed to downregulate ChAT expression and an ACh level in the heart, unlike ChATKD mice. Mice overexpressing only one ChAT siRNA showed comparable cardiac ACh levels and expression levels of glucose metabolism-related protein with WT mice. Transgene expression was verified to be solely detected in the heart by the heart-specific green fluorescent protein (GFP) expression (Fig. 1a) because siRNAs were not translated to proteins.

The experimental protocols in this study were approved by the Animal Care and Research Ethics Committee of Nippon Medical School in Tokyo, Japan (permission number: 27-003). All animal experiments were performed in strict accordance with the recommendations set by the Directive 2010/63/EU of the European Parliament and the National Institutes of Health guide for the care and use of laboratory animals. Animal stress and suffering were minimized as much as possible. Both male ChATKD and age-matched control mice were randomly assigned to experimental groups, and the experimenters were performed without their genotype’s information. For euthanasia, the mice were subjected to cervical dislocation.

#### Transgenic mice with the heart-specific overexpression of ChAT gene (ChAT tg)

As previously reported, we bred ChAT tg mice and maintained their population by mating them with wild-type (WT) mice. Male ChAT tg mice were already identified to have comparable cardiac function and hemodynamic parameters with WT mice [9]. We used ChAT tg mice as an opposite reference in comparison with ChATKD mice.

### Evaluation of expression levels in mRNA, protein and ACh in the heart

#### Measurement of ACh in the heart

Based on our previous report [1, 13], cardiac ventricles without the atria (100 mg tissue) were homogenized in a specific lysis buffer for ACh measurement. The buffer (1 mL) included 0.1 M of perchloric acid, 0.1 mM of physostigmine, 0.1 mM of EDTA and an isopropyl homocholine internal control (1 × 10^–8^ M, Eicom Corp., Kyoto, Japan). After centrifugation, the pH of the supernatant was increased to more than 6.5 using 1 M KHCO_3_, followed by purification with 0.5 mL Amicon® Ultra column centrifuge filter (10 K, Merck Millipore, Tokyo, Japan). Some of the flow-through collected (10 μL) was introduced into a high-performance liquid chromatography (HPLC) system (HTEC-500, Eicom, Kyoto, Japan) to measure a cardiac ACh content (M/ 0.1 g tissue weight).

#### qRT-PCR

qRT-PCR was conducted according to our previous experiment [13]. Total RNA (0.5 μg) was isolated from the heart with ISOGEN II (Nippon Gene Co., Ltd., Tokyo, Japan), and reverse-transcribed with ReverTra Ace® qPCR RT Master Mix (Toyobo Life Science, Osaka, Japan), followed by qPCR with GoTaq® qPCR Master Mix (Promega KK, Tokyo, Japan) using Thermal Cycler Dice® Real Time System TP800 (Takara Bio Inc., Shiga, Japan). The relative expression levels of all interested mRNAs were quantified to the levels of β-actin mRNA. The specific primers for murine atrial natriuretic peptide (ANP), brain natriuretic peptide (BNP), ChAT, and β-actin were prepared, as follows,

ANP (forward), CAGCATGGGCTCCTTCTCC;

ANP (reverse), TCCGCTCTGGGCTCCAATCCT;

BNP (forward), CTGAAGGTGCTGCCCCAGATG;

BNP (reverse), GACGGATCCGATCCGGTC;

ChAT (forward), TGGATGAAACATACCTGATGAGCAA;

ChAT (reverse), CGTGAAAGCTGGAGATGCAGAA;

β-actin (forward), CATCCGTAAAGACCTCTATGCCAAC; and β-actin (reverse), ATGGAGCCACCGATCCACA.

For the other analysis of gluconeogenesis-related mRNA levels, i.e., phosphoenolpyruvate carboxykinase (PEPCK) and glucose-6-phosphatase (G6Pace), in the liver, the following murine primers were used:

PEPCK (forward), ACTGTTGGCTGGCTCTCACT;

PEPCK (reverse), TGCAGGCACTTGATGAACTC;

G6Pase (forward), CCAGTCGACTCGCTATCTCC; and

G6Pace (reverse), TGGCTTTTTCTTTCCTCGAA.

#### Western blot analysis

According to previous studies [1, 14], 10–30 μg protein in each lane, which was isolated from the murine cardiac ventricles, processed for quantification, and mixed with a sampling buffer (1 × : 0.05 M Tris–HCL pH 6.8, 0.1 M DTT, 0.07 M SDS, 0.1% (W/V) bromophenol blue, 6% (v/v) glycerol, and 2.5% (v/v) 2-mercaptoethanol), was comparably electrophoresed in 10% or 15% of SDS-PAGE gel and blotted into a polyvinylidene difluoride (PVDF) membrane. It was reacted with a primary antibody, followed by an HRP-conjugated secondary antibody; signals were detected with a C-DiGit Blot Scanner (LI-COR Corp., Lincoln, NE, USA) using an ImmunoStar® LD (Wako Pure Chemical Industries, Ltd., Tokyo, Japan). Each Western blot analysis was repeated at least three times to evaluate the consistency. Primary antibodies used in this study were as follows:

a) For analysis of cell signaling: anti-HIF-1α antibody (ab) (1:500, Novus Biologicals, Littleton, Co, USA), and anti-phospho-Akt and anti-Akt abs (1:500–1:1000, Cell Signaling Technology, Danvers, MA, USA);

b) For analysis of glucose metabolism and energy metabolism: anti-Glut-4 ab (1:2000, Merck Millipore, Darmstadt, Germany), anti-PDK4 ab (1:1000, Novus Biologicals), anti-PGC-1α ab (1:2000, Merck Millipore), anti-PEPCK ab (1:1000, Santa Cruz Biotechnology Inc., Dallas, TX, USA), G6Pase ab (1:1000, Bioss Antibodies Inc., Woburn, MA, USA), and anti-Sirt6 ab (1:1000, Novus Biologicals);

c) For analysis of cardiac functions: anti-VEGF ab (1:1000, Santa Cruz Biotechnology, CA, USA), anti-ChAT ab (1:2000, Millipore, Billerica, MA, USA), anti-ANP ab (1:1000, Santa Cruz Biotechnology), anti-nitrotyrosine ab (1:2000, Santa Cruz Biotechnology Inc.), and anti-NOS1 or NOS3 ab (1:1000, Santa Cruz Biotechnology);

d) For miscellaneous analysis: anti-GFP ab (1:1000, Medical & Biological Laboratories, Co., Ltd, Nagoya, Aichi, Japan), anti-β-actin ab (1:2000, Santa Cruz Biotechnology Inc.) and anti-GAPDH ab (1:3000, Santa Cruz Biotechnology Inc.).

### Cardiac function analysis

According to a previous study [14], male mice were anesthetized with isoflurane at a flow rate of 0.5–1.0 L/min (NARCOBIT, Natsume Seisakusho, Co., Tokyo, Japan), and we compared the cardiac function of the anesthetized male ChATKD and age-matched control mice, using an admittance PV catheter (1.2F; Transonic Systems Inc., Ithaca, NY, USA) inserted via the apical approach under artificial ventilation conditions. The catheter was connected to an ADVantage PV system control box with a data acquisition system (Transonic Systems Inc.). All hemodynamic parameters were measured and recorded using the Lab-Scribe2 software (iWorx Systems), including heart rate (HR), end-systolic pressure (ESP), end-diastolic pressure (EDP), end-systolic volume (ESV), end-diastolic volume (EDV), stroke volume (SV), cardiac output (CO), and ejection fraction (EF) [14]. After measuring the cardiac function followed by cervical dislocation, the organs were excised for sample collection. When samples were collected without cardiac function evaluation, the mice were sacrificed only by cervical dislocation.

### Morphological analysis

#### Masson’s trichrome staining

According to the previous study [9], a paraffin-embedded heart section (4 μm thickness) was deparaffinized, dehydrated and stained with Masson’s trichrome stain solution. Fibrotic changes displayed blue-colored stain, and the muscles showed pink.

#### Immunohistochemical analysis

As our previous studies demonstrated [9, 10, 14], immunohistochemical studies were performed with a paraffin-section of the heart, using the Ventana automated immunohistochemistry system (Discovery TM, Ventana Medical System, Inc., Tucson, AZ, USA). Antigen retrieval was performed for 60 min in a preheated Dako Target Retrieval Solution (pH 6.0) using a microwave, followed by intrinsic peroxidase inhibition, blocking, and reaction with an anti-VEGF ab (1:100, Santa Cruz Biotechnology) or anti-von Willebrand factor ab (1:100, Dako, Glostrup, Denmark). The antigen was then reacted with a reaction with a biotin-conjugated anti-rabbit IgG ab, based on the streptavidin–biotin–peroxidase reaction with 3,3′-diaminobenzidine (DAB). Counter staining of the nuclei was conducted with hematoxylin stain.

#### Immunofluorescence analysis

After 4% paraformaldehyde (PFA) perfusion fixation followed by immersion fixation, the heart was embedded in an Optimal cutting temperature (OCT) compound and sectioned. After permeabilization with 0.1% Triton-X, the sections were double reacted with primary antibodies including a rabbit anti-NOS1 ab (1:100, Abcam, Tokyo, Japan), anti-CD34 ab (1:200, Abcam), mouse anti-ryanodine receptor (RYR) ab (1:100, Abcam), followed by a goat anti-rabbit IgG ab (Alexa Fluor®568 or 488) or a donkey anti-mouse IgG ab (Alexa Fluor®488), respectively. The nuclei were stained using DAPI. Finally, they were observed with Confocal Laser Scanning Microscope FV3000 (Olympus Corp. Tokyo, Japan).

### Analysis of functions of NO, ROS, and calcium handling in the heart

#### Tissue NO_2_ concentration measurement in the heart

Based on the previous study [14], the cardiac NO_2_ levels were measured using the QuantiChrom™ Nitric Oxide Assay Kit (BioAssay Systems, Hayward, CA, USA). Excised hearts (100 mg) were homogenized in 300 μL of phosphate-buffered saline (PBS), and the assay was conducted following the manufacturer’s instructions. The final concentration of NO_2_ was determined by OD at 540 nm absorbance with reference to the predetermined NO_2_ levels (μM).

#### Immunoprecipitation of the heart lysates to evaluate protein interaction in NOS1, RYR, and smooth endoplasmic reticulum Ca^2+^ ATPase (SERCA) 2a

To investigate the interaction between RYR and NOS1 or SERCA2a, the cardiac ventricles of the ChATKD or control mice were excised and homogenized in a lysis buffer. After centrifugation, the supernatants, which were quantified to be comparable in protein contents, were treated with magnet beads alone without conjugated antibody in room temperature. Afterwards, beads conjugated with anti-RYR ab, prepared by mixing each other with rotation at room temperature 1 h before, were added to the precleared supernatant, followed by 4 h rotation at 4 °C. After washing three times with PBS, the immunoprecipitated proteins were mixed with a sampling buffer, and boiled. Equal volumes of the immunoprecipitated samples were electrophoresed and blotted to a membrane. Subsequently, the membrane was reacted with a primary antibody including anti-NOS1 or anti-SERCA2a ab, followed by HRP-conjugated secondary IgG ab. The signals were detected by a C-DiGit Blot Scanner (LI-COR Corp), using ImmunoStar® LD (Wako Pure Chemical Industries).

#### Evaluation of ROS-related analysis by superoxide dismutase (SOD) activity, malondialdehyde (MDA) level, nitrotyrosine immunoreactivity, and γ-H2A protein expression level

SOD activities in the hearts of control and ChATKD mice were measured using the SOD assay kit, according to the manufacturer’s instruction (Dojindo Laboratories, Kumamoto, Japan). Briefly, the heart was homogenized in a buffer including 0.25 M sucrose, 10 mM Tris–HCl (pH 7.4), and 1 mM EDTA. After centrifugation at 10,000 ×*g* for 60 min, the supernatant was obtained as a sample. The sample, which was sequentially diluted and mixed with WST working solution, was incubated at 37 °C for 20 min, and then, a sample OD at 450 nm was measured. From 50% inhibition rate, SOD activity was evaluated. The lipid peroxidation levels were measured using MDA assay kit (Abcam, Tokyo, Japan) under the manufacturer’s instruction.

A western blot analysis was performed according to the protocol mentioned above using an anti-phospho-Histone H2A.X ab (1:2000, Santa Cruz Biotechnology) and anti-nitrotyrosine ab (1:2000, Santa Cruz Biotechnology Inc.), followed by HRP-conjugated anti-mouse IgG ab.

#### Measurement of an ATP level in the heart

According to the manufacture’s instruction, ATP levels were measured to be compared between control and ChAT KD mice. Whole hearts, whose weights were comparably adjusted, were excised and homogenized in a lysis buffer, and then an ATP isolation reagent was added. Afterward, the samples were mixed with an ATP luminescence regent and measured by a luminometer.

### Whole animal analysis of depressive-like phenotypes

#### Blood corticosterone level measurement

According to our previous study [14], the blood was collected from mice by cardiac puncture to measure the corticosterone concentration using the corticosterone ELISA Kit (Cayman Chemical Company, Ann Arbor, MI, USA) in the morning (before 10 AM).

#### Tail suspension test (TST) and forced swim test (FST)

As reported in the previous study [14], TST and FST were performed in the ChAT KD and control mice to evaluate their depression-like conditions while measuring an immobility time during the tests. In TST, the time of being immobile was measured during 10 min duration. In contrast, in FST, the time spent immobile was measured only during the last 4 min of the test.

#### Resident-intruder aggression tests

Experimental subjects underwent one test in the transparent cages. Intruders were adult C57BL/6J male mice and had same body weight as the resident. An aggression test was begun when the intruder was transferred into the resident cage and all events during the test were recorded in a digital recording medium. The two mice were recorded for the initial 10 min, during which the resident attacked the intruder. The total attacking time for each pair was measured and the attacking rate was evaluated.

### Vagus nerve activity measurement

According to the previous study [14], the vagus nerve activity was measured. A pair of silver wire electrodes were attached to the left cervical vagus nerve. The vagus nerve electrical activity was amplified 1000 times with a band path filter of 10 Hz–1 kHz. While monitoring with an oscilloscope, the vagal neurogram was recorded and the nerve activity parameters were evaluated using the Mini Analysis Program (Synaptosoft, Inc. Decatur, GA, USA).

### Statistical analysis

Data were expressed as mean ± standard error of mean (SEM). Comparison between the two groups was conducted with the unpaired non-parametric Mann–Whitney U-test for analysis of western blot data; other than those, unpaired t-test was used. For multiple comparisons, the differences among groups were assessed using non-repeated ANOVA, followed by SNK test. The attacking rate differences between the age-matched control and ChATKD mice were compared using the Chi-square test. The differences were evaluated to be significant at a *P* value of < 0.05.

## Results

### ChATKD hearts show the heart-specific transgene expression and the ChAT mRNA expression downregulation

Western blot analysis using anti-GFP antibodies revealed that the transgene was restrictively expressed in the heart (Fig. 1a) because the IRES sequence enabled the simultaneous EmGFP and siRNA gene expressions for the ChAT downregulation. However, cardiac GFP expression levels of ChATKD mice were not altered, but comparable with those of ChAT tg mice possessing better cardiac function (0.92 ± 0.04 vs. 1.06 ± 0.06, *n* = 4).

Cardiac ChAT mRNA expression was lower in the ChATKD mice than in the control mice (0.0054 ± 0.0027 vs. 1.52 ± 0.68, *t* = 2.213, *P* = 0.001, *n* = 4–10; Fig. 1b). Similarly, cardiac ChAT protein expression was lower in the ChATKD mice (Fig. 1c). Therefore, cardiac ACh content was lower in the ChATKD mice than in the control mice (1.89 ± 0.33 (× 10^–8^) vs. 3.72 ± 0.37 (× 10^–8^), *t* = 3.69, *P* = 0.001, *n* = 9; Fig. 1d). Hence, the hearts of the ChATKD mice possessed a downregulated NNCCS.

### ChATKD mice show reduced blood pressure

**Fig. 2:**
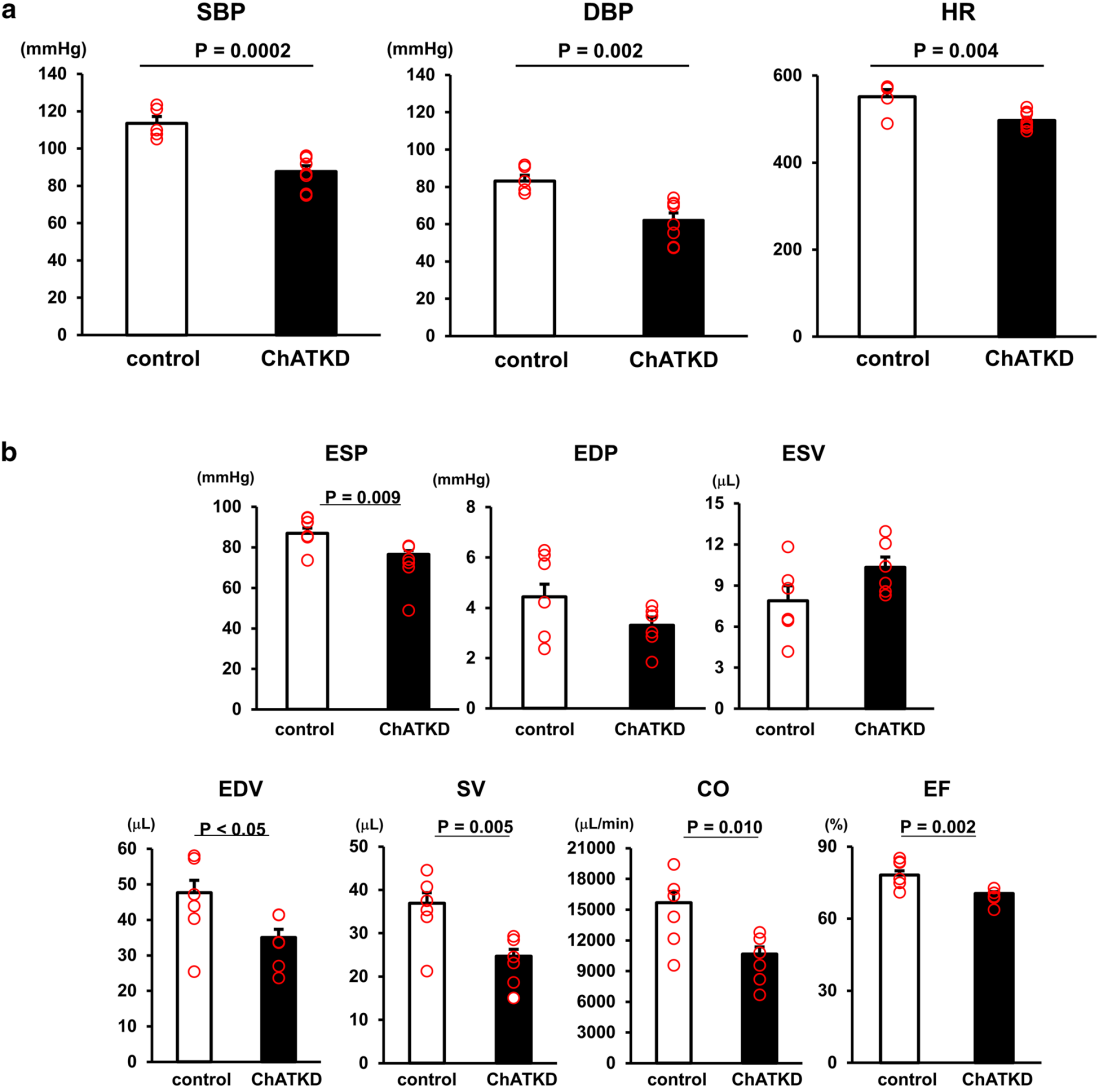
ChATKD hearts show impaired cardiac function, compatible with heart failure, accompanied with depressed hemodynamic parameters. **a** SBP (*P* = 0.0002), DBP (*P* = 0.002), and HR (*P* = 0.004) are lower in the ChATKD mice than in control mice. **b** ESP (*P* = 0.009), SV (*P* = 0.005), CO (*P* = 0.010), and EF (*P* = 0.002) are lower at 4 months of age in ChATKD mice than in control mice

The systolic (87.7 ± 3.1 mmHg vs. 113.5 ± 3.7 mmHg, *t* = 5.38, *P* = 0.0002, *n* = 5–8) and diastolic (62.1 ± 3.9 mmHg vs. 83.1 ± 3.1 mmHg, *t* = 4.43, *P* = 0.002, *n* = 5–8) pressures and the heart rate (497 ± 7 vs. 552 ± 16 beats/min, *t* = 3.11, *P* = 0.004, *n* = 5–8) were lower in the conscious ≥ 4 months aged ChATKD mice than in the control mice (Fig. 2a). The reduction of HR was different from the typical phenotype of HR upregulation in heart failure.

### Cardiac function is impaired specifically after growth in the ChATKD mice compared with the control mice

Our cardiac function analysis at 4 months of age showed that ESP, SV, EF, and CO were significantly lower in the ChATKD mice than in age-matched control mice (ESP: 76.4 ± 1.9 mmHg vs. 86.9 ± 2.6 mmHg, *t* = 2.81, *P* = 0.009; SV: 24.7 ± 1.6 μL vs. 36.9 ± 2.4 μL, *t* = 3.10, *P* = 0.005; CO: 10,672.0 ± 683.8 μL/min vs. 15,679.6 ± 1084.2 μL/min, *t* = 2.75, *P* = 0.010; EF: 70.5 ± 0.7% vs. 78.2 ± 1.7%, *t* = 3.81, *P* = 0.002, *n* = 6 in each parameter; Fig. 2b), suggesting that the ChATKD mice suffer from cardiac dysfunction.

### ChATKD hearts upregulate several molecular markers related to cardiac dysfunction

**Fig. 3:**
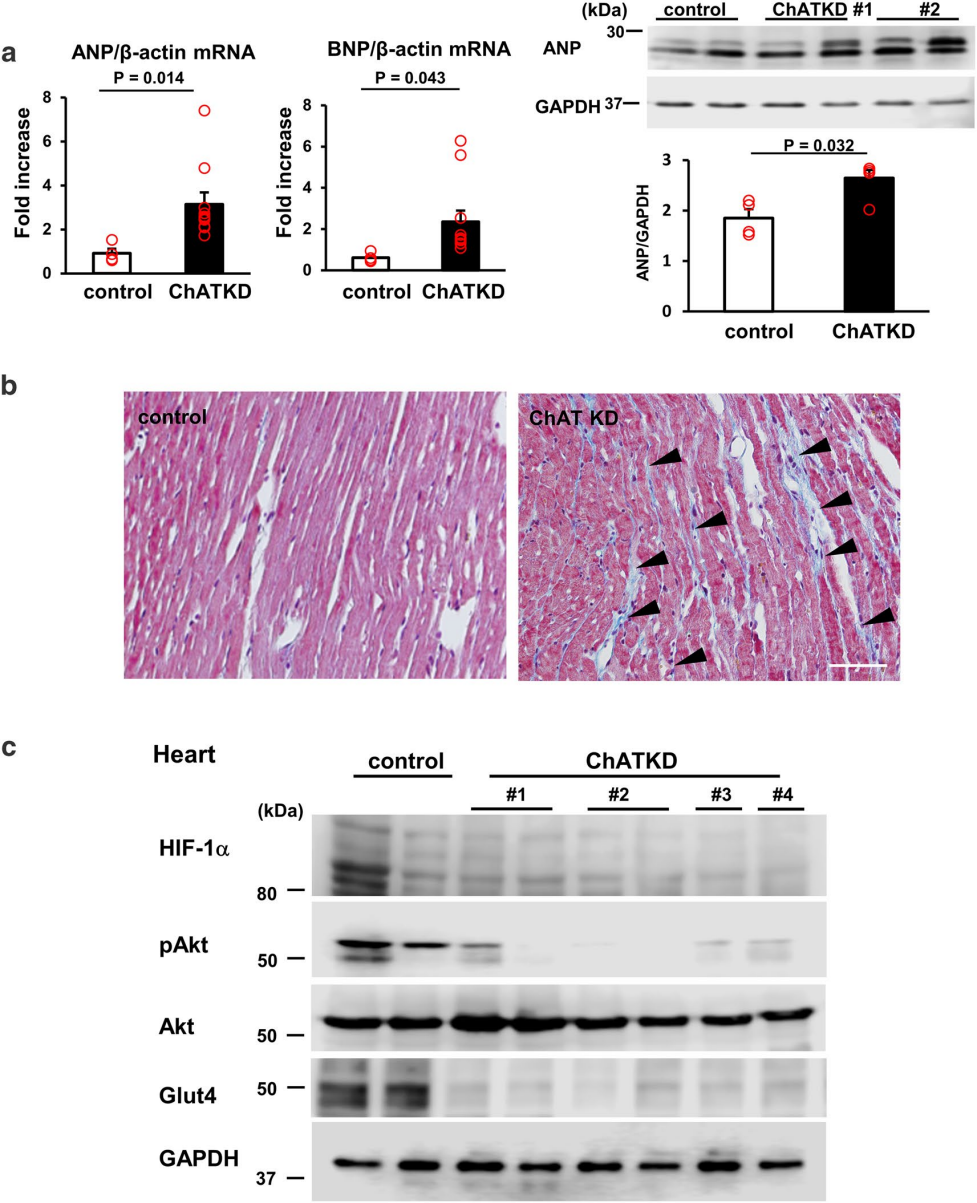

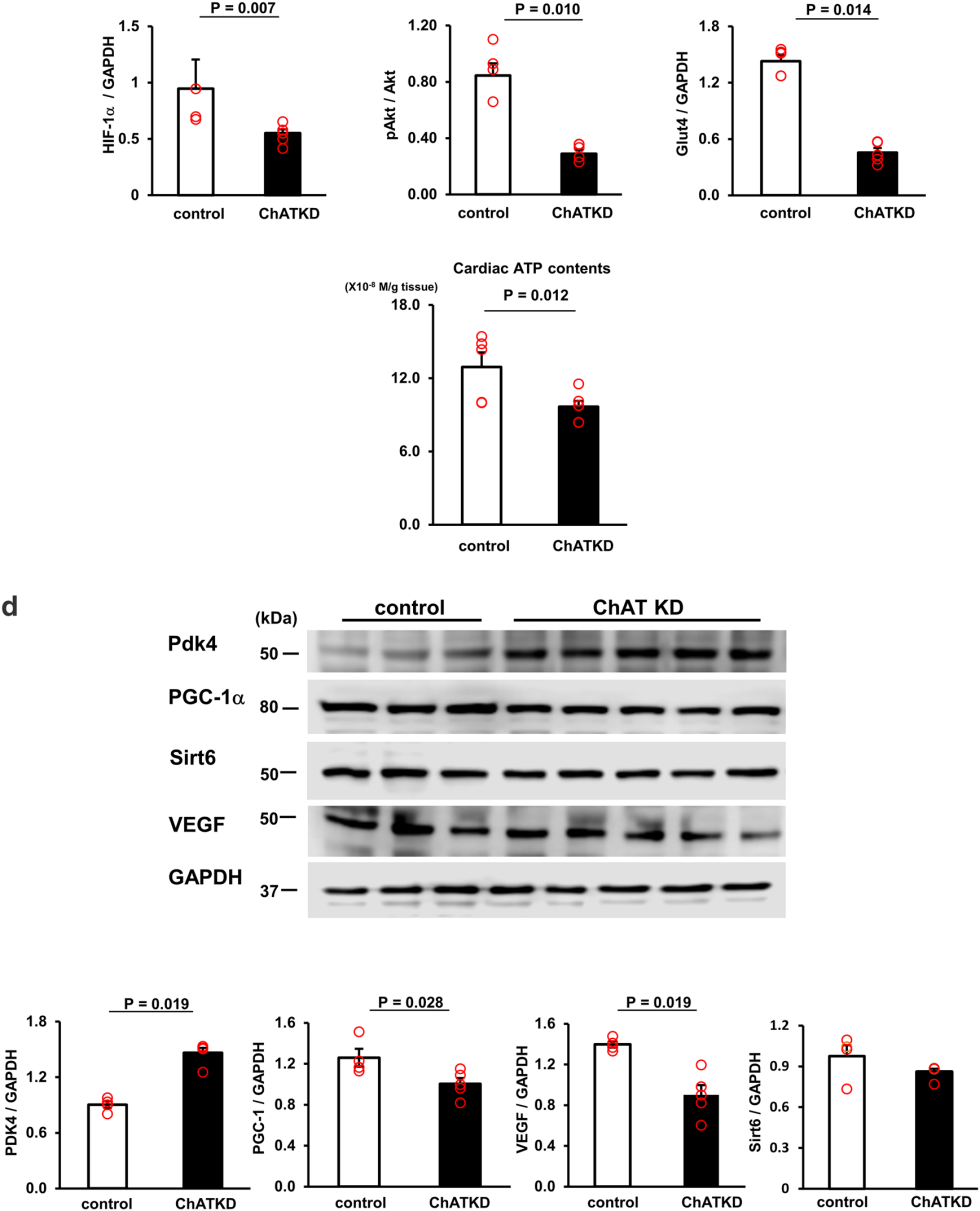

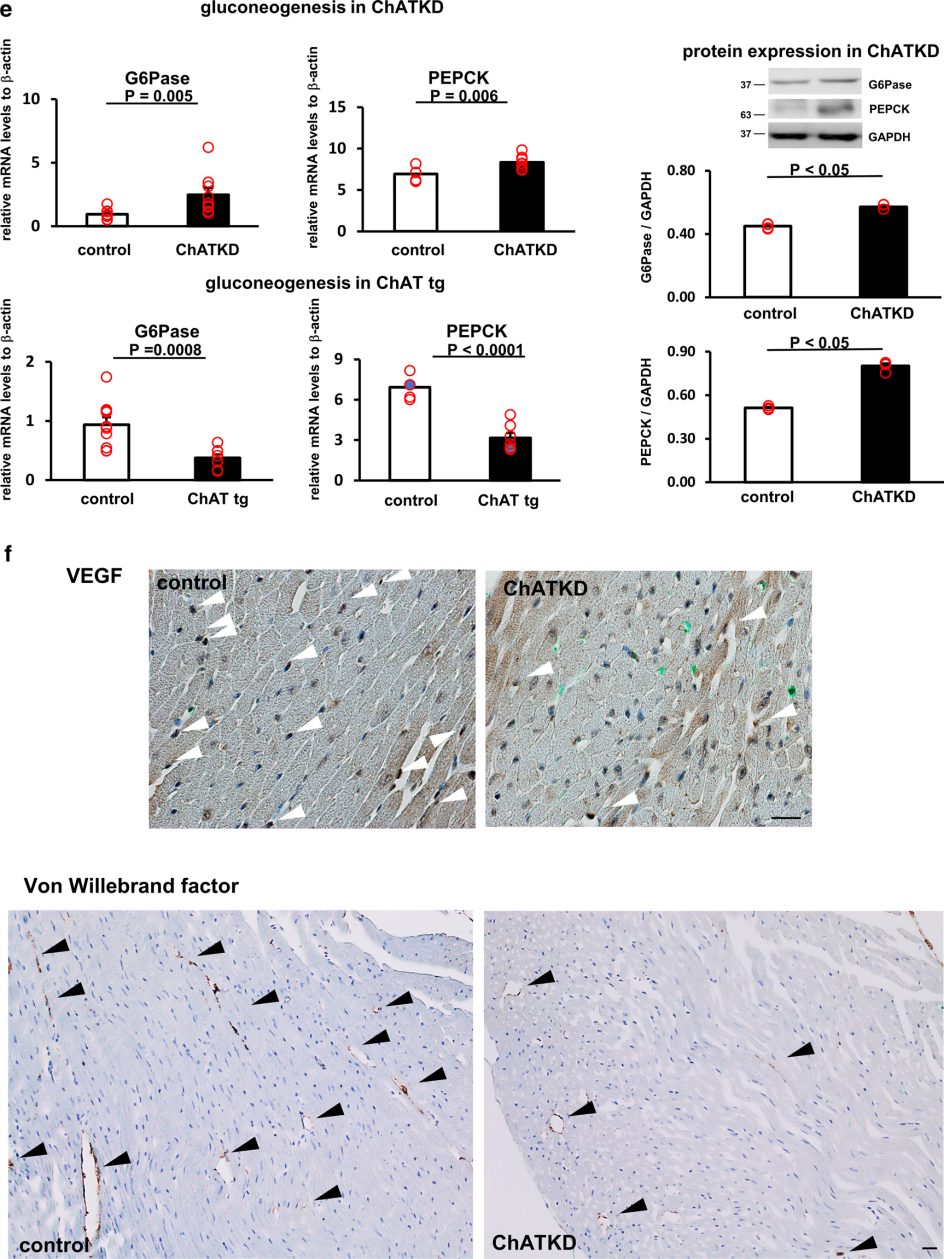

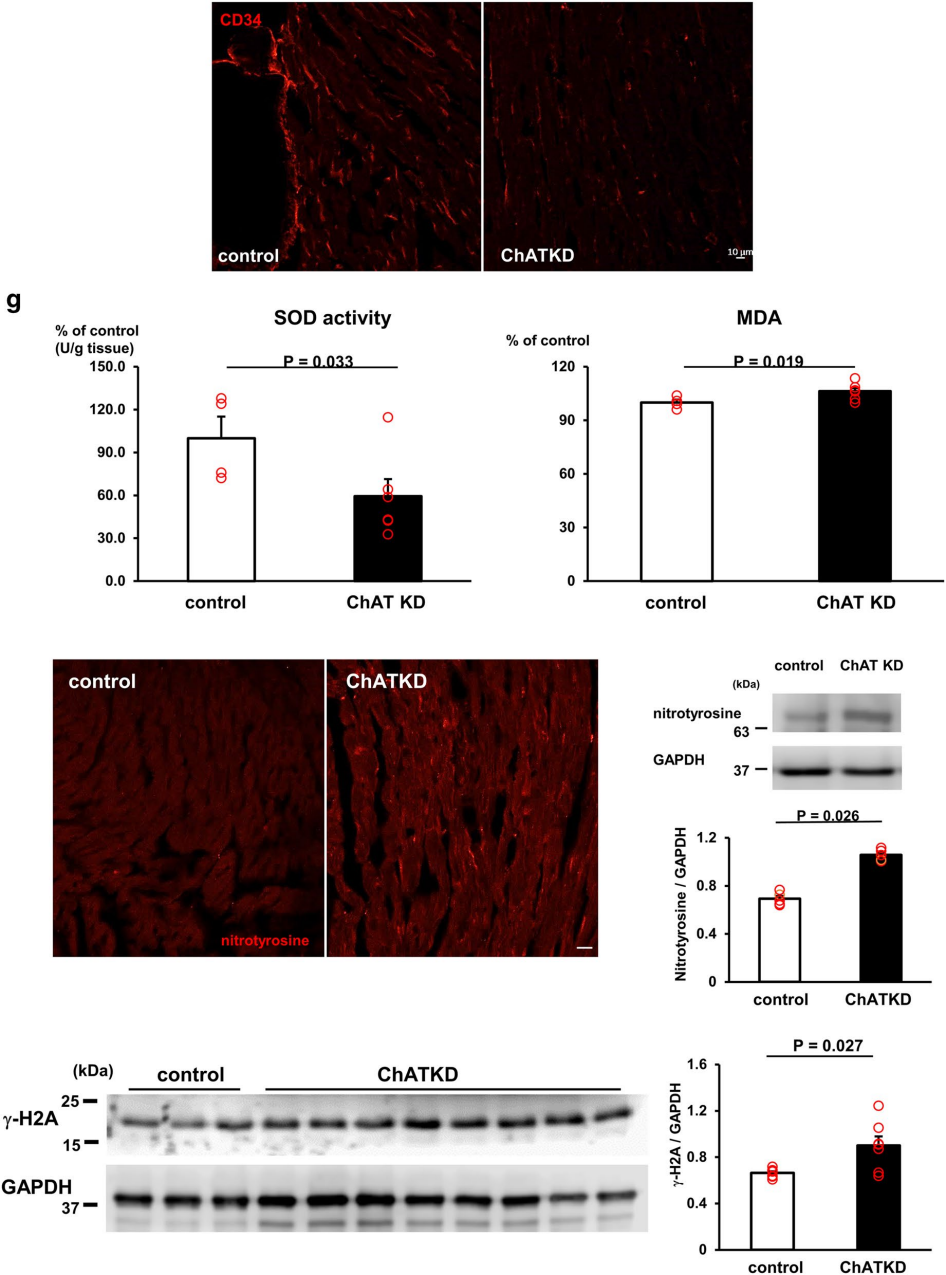
ChATKD hearts show an elevation in heart failure markers, morphological changes in the ventricle, and enhanced reactive oxygen species with DNA damage. **a** The ANP and BNP mRNA levels are both increased in ChATKD hearts (*P* = 0.014 and 0.043, respectively). Furthermore, the ANP protein expression levels are upregulated in the ChATKD hearts (*P* = 0.032). **b** Masson’s trichrome staining displays the ChATKD hearts possessing characteristic fibrosis (black arrowheads) in the ventricles. Scale bar: 50 μm. **c** The expression levels of glucose utilization-related factors, i.e., HIF-1α (*P* = 0.007), pAkt/Akt (*P* = 0.010), and Glut4 (*P* = 0.014) are lower in the ChATKD hearts than in the control hearts. Cardiac ATP levels are lower in ChATKD mice than in control mice (*P* = 0.012). **d** Reciprocally, the PDK4 expression levels are higher in the ChATKD hearts (*P* = 0.019); in contrast, the PGC-1 (*P* = 0.028) and VEGF levels (*P* = 0.019) are lower in the ChATKD hearts. **e** ChATKD mice upregulate gluconeogenesis in the liver. Hepatic mRNA levels of G6Pase (*P* = 0.005) and PEPCK (*P* = 0.006) are higher in ChATKD mice than in control mice, and each protein expression also shows the same increasing trend (*P* < 0.05); however, this is in contrast to the ChAT tg mice, which reciprocally downregulate gluconeogenesis in the liver (G6Pase, *P* = 0.0008; PEPCK, *P* < 0.0001). **f** VEGF immunoreacted endothelial cells (white arrowheads) appear reduced in the ChATKD hearts. Immunoreactivity of von Willebrand factor (black arrowheads) is attenuated in ChATKD hearts. Scale bar: 50 μm. CD34 signal levels in the heart are also attenuated in ChATKD mice compared with those in control mice. Scale bar: 10 μm. **g** ChATKD hearts are more exposed to augmentation of the ROS production through MDA (*P* = 0.019), nitrotyrosine stain intensity associated with the increased protein expression levels (*P* = 0.026), and γ-H2A expression (*P* = 0.027), but reciprocally attenuated SOD activity (*P* = 0.033), than the control hearts. Scale bar: 20 μm

To further check whether ChATKD mice were subjected to cardiac dysfunction, the levels of molecular marker for cardiac dysfunction were examined. In Fig. 3a, the ANP (3.14 ± 0.54 vs. 0.91 ± 0.21, *t* = 3.83, *P* = 0.014, *n* = 4–9) and BNP (2.35 ± 0.55 vs. 0.61 ± 0.11, *t* = 3.10, *P* = 0.043, *n* = 4–9) mRNA expression levels, as well as ANP protein levels (2.64 ± 0.16 vs. 1.85 ± 0.18, *P* = 0.032, *n* = 4–8), were significantly higher in ChATKD hearts than in control hearts, revealing that the ChATKD heart function was impaired. Compatible with these, more fibrotic changes, as evidenced by Masson’s trichrome staining, was higher in ChATKD hearts (Fig. 3b; black arrowheads: fibrotic changes).

### ChATKD hearts show altered molecular marker expression responsible for cardiac energy metabolism

Intriguingly, ChATKD hearts possessed reduced protein expression of HIF-1α, pAkt and Glut4 (HIF-1α: 0.55 ± 0.03 vs. 0.95 ± 0.16, *P* = 0.007; pAkt/Akt: 0.29 ± 0.03 vs. 0.85 ± 0.09, *P* = 0.010; Glut4: 0.46 ± 0.05 vs. 1.43 ± 0.06, *P* = 0.014, *n* = 5–6 in each marker; Fig. 3c). These patterns suggest that factors related to glucose metabolism in the heart may be impaired in ChATKD mice because HIF-1α is a master transcription factor for the process. The ATP levels of ChATKD hearts were decreased compared with those of control hearts (9.7 ± 0.5 vs. 12.9 ± 1.2 (× 10^–8^ M/g tissue), *t* = 2.52, *P* = 0.012, *n* = 4–6, Fig. 3c).

The cardiac expression of PDK4 was higher in the ChATKD mice than in the control mice (1.46 ± 0.05 vs. 0.90 ± 0.04, *P* = 0.019, *n* = 4–5; Fig. 3d), suggesting that the transition from glycolysis to the tricarboxylic acid (TCA) cycle was disturbed because PDK4 is a negative regulator of pyruvate dehydrogenase (PDH) promoting efficient pyruvate entrance into the TCA cycle. Protein expression of PGC-1, involved in fatty acid oxidation, was significantly more depressed in ChATKD hearts (1.00 ± 0.06 vs. 1.26 ± 0.09, *P* = 0.028, *n* = 4–5; Fig. 3d). The Sirt6 in ChATKD showed a comparable expression (0.86 ± 0.02 vs. 0.98 ± 0.08, *n* = 4). Based on this impaired cardiac glucose utilization of the ChATKD heart, imbalanced glucose responses in the ChATKD livers were suggested (Fig. 3e). mRNA expression levels of gluconeogenesis responsible genes, G6Pase (2.47 ± 0.54 vs. 0.94 ± 0.12, *t* = 2.76, *P* = 0.005, *n* = 9–10) and PEPCK (8.34 ± 0.29 vs. 6.92 ± 0.39, *t* = 2.97, *P* = 0.006, *n* = 5–8) were higher in ChATKD livers than in control livers, accompanied with similarly increased patterns of G6Pase (0.57 ± 0.01 vs. 0.45 ± 0.01, *P* < 0.05, *n* = 4) and PEPCK (0.80 ± 0.02 vs. 0.51 ± 0.01, *P* < 0.05, *n *= 4) protein levels in ChATKD livers. These mRNA alterations in ChATKD livers were in contrast to the ChAT tg livers showing rather reciprocal changes (G6Pase: 0.37 ± 0.06 vs. WT, *t* = 4.09, *P* = 0.0008, *n* = 8–10; PEPCK: 3.16 ± 0.37 vs. WT, *t* = 6.99, *P* < 0.0001, *n* = 5–7; Fig. 3e).

VEGF protein expression was reduced in the ChATKD mice (0.90 ± 0.10 vs. 1.40 ± 0.03, *p* = 0.019, *n* = 4–5) and the reduction was supported by immunohistochemical and immunofluorescent staining (Fig. 3d and f). DAB positive VEGF signal positive endothelial cells were decreased in the ChATKD mice. Moreover, signals of von Willebrand factor and CD34 were both decreased in ChATKD, compared to those in control hearts (Fig. 3f).

### ChATKD hearts show depressed SOD activity and accelerated ROS generation

The SOD activity, one of the self-defense molecules against ROS in the heart, was measured and compared between ChATKD mice and control mice. As shown in Fig. 3g, the activity levels were significantly lower in ChATKD hearts (59.3 ± 12.0 vs. 100.0 ± 15.1, *t* = 2.109, *P* = 0.033, *n* = 4–6). In contrast, the MDA levels, a marker for ROS, were higher in the ChATKD mice (106.3 ± 1.7% vs. 100 ± 1.6%, *t* = 2.67, *P* = 0.019, *n* = 4–7). The nitrotyrosine immunoreactivity and γ-H2A protein expression levels (0.90 ± 0.08 vs. 0.66 ± 0.01, *P* = 0.027, *n* = 7), both ROS-related markers, were higher in the ChATKD heart. Nitrotyrosine protein expression levels were also increased in ChATKD hearts (1.06 ± 0.02 vs. 0.69 ± 0.02, *n* = 5, *P* = 0.026) (Fig. 3g). Altogether, it was clearly indicated that ChATKD hearts were subjected to the exaggerated ROS burden, leading to accelerated DNA damage, and reciprocally attenuated anti-ROS machinery.

### ChATKD hearts show a failure to synthesize nitric oxide (NO) and possess reduced vagus nerve activity

**Fig. 4:**
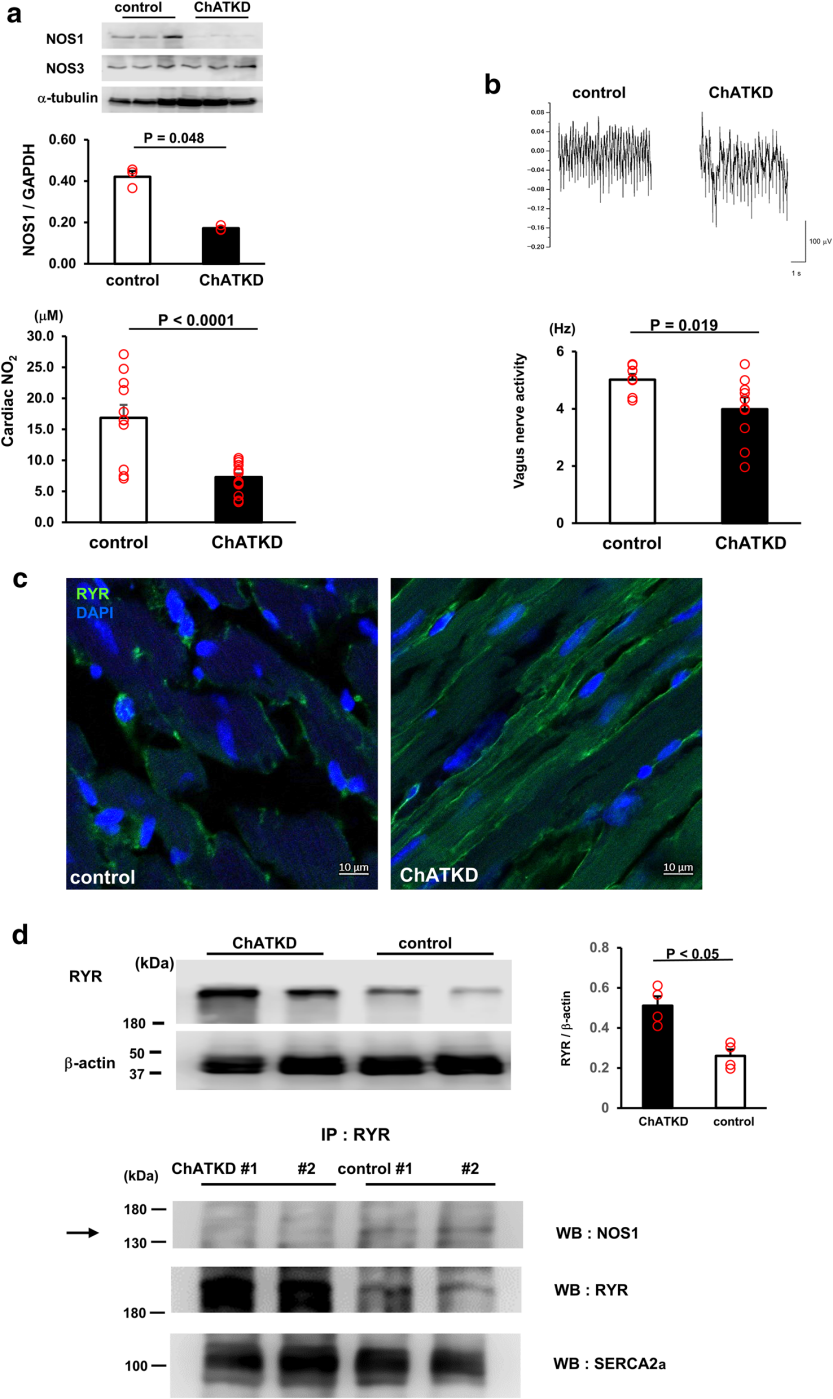
Attenuation of cardiac NO synthesis potential and vagus nerve activity with altered ryanodine receptor expression in the ChATKD hearts. **a** A NO metabolite, NO_2_, in the hearts is significantly lower in ChATKD hearts than in WT hearts (*P* < 0.0001) due to depressed NOS1 (*P* = 0.048) but not NOS3 expressions in the hearts. **b** Neuronal activity of the vagus nerve is lower in ChATKD mice than in control mice (*P* = 0.019). **c** Immunoreactivity of ryanodine receptor (RYR) is exclusively more enhanced in cytoplasm of the cardiomyocytes in ChATKD hearts than in control hearts. Scale bar: 10 μm. **d** RYR, whose protein expression is enhanced in ChATKD hearts, and NOS1 interaction (arrow) is impaired in the ChATKD hearts

To examine potency for NO production in ChATKD heart, the NO_2_ contents of the hearts, NO metabolites, were measured. Surprisingly, the NO_2_ levels in ChATKD hearts were significantly reduced to 50% of those in control hearts (8.1 ± 0.5 μM vs. 16.8 ± 2.1 μM, *t* = 4.08, *P* < 0.0001, *n* = 11–25) (Fig. 4a). This was further supported by depressed cardiac NOS1 protein expression (0.17 ± 0.01 vs. 0.42 ± 0.03, *P* = 0.049, *n* = 3) without NOS3 level alteration [2, 30, 31], suggesting that cardiac NO synthesis is impaired in ChATKD mice (Fig. 4a).

Our previous study using heart-specific ChAT transgenic mice (ChAT tg) reported activated cardiac NO synthesis, which was involved in the characteristic central phenotypes through the increased vagus nerve activity because a NOS inhibitor blunted the beneficial phenotypes [14]. In contrast, in ChATKD mice, the vagus nerve activity represented by its frequency was decreased (4.0 ± 1.5 Hz vs. 5.0 ± 1.9 Hz in WT, *t* = 2.56, *P* = 0.019, *n* = 7–10, Fig. 4b). This suggests that impaired ascending signals of the vagus nerve and attenuated NO release from the ChATKD heart may be involved not only in the progression of cardiac dysfunction, but also in a ChATKD CNS phenotype, one of extra-cardiac phenotype.

Intriguingly, the ryanodine receptor (RYR) signals were reciprocally more accentuated in ChATKD hearts than in WT hearts (Fig. 4c), and it was evidenced by increased RYR protein expression levels in ChATKD compared with those in control mice (0.51 ± 0.05 vs. 0.26 ± 0.03, *P* < 0.05, *n* = 4) suggesting a possible disturbed interaction between the NOS1 and RYRs during the attenuation of the cardiac ChAT expression. Our immunoprecipitation experiment revealed that cardiac ventricles-derived homogenates, immunoprecipitated with an anti-RYR antibody, had a lower NOS-1 protein expression in ChATKD hearts than in control hearts (Fig. 4d). Taken together, ChATKD hearts possessed attenuated interaction between the NOS-1 and RYR in their localization.

### Evaluating the influences of NNCCS on the central nervous system using ChATKD mice

#### ChATKD hearts possess an elevated blood corticosterone level in the basal condition

**Fig. 5:**
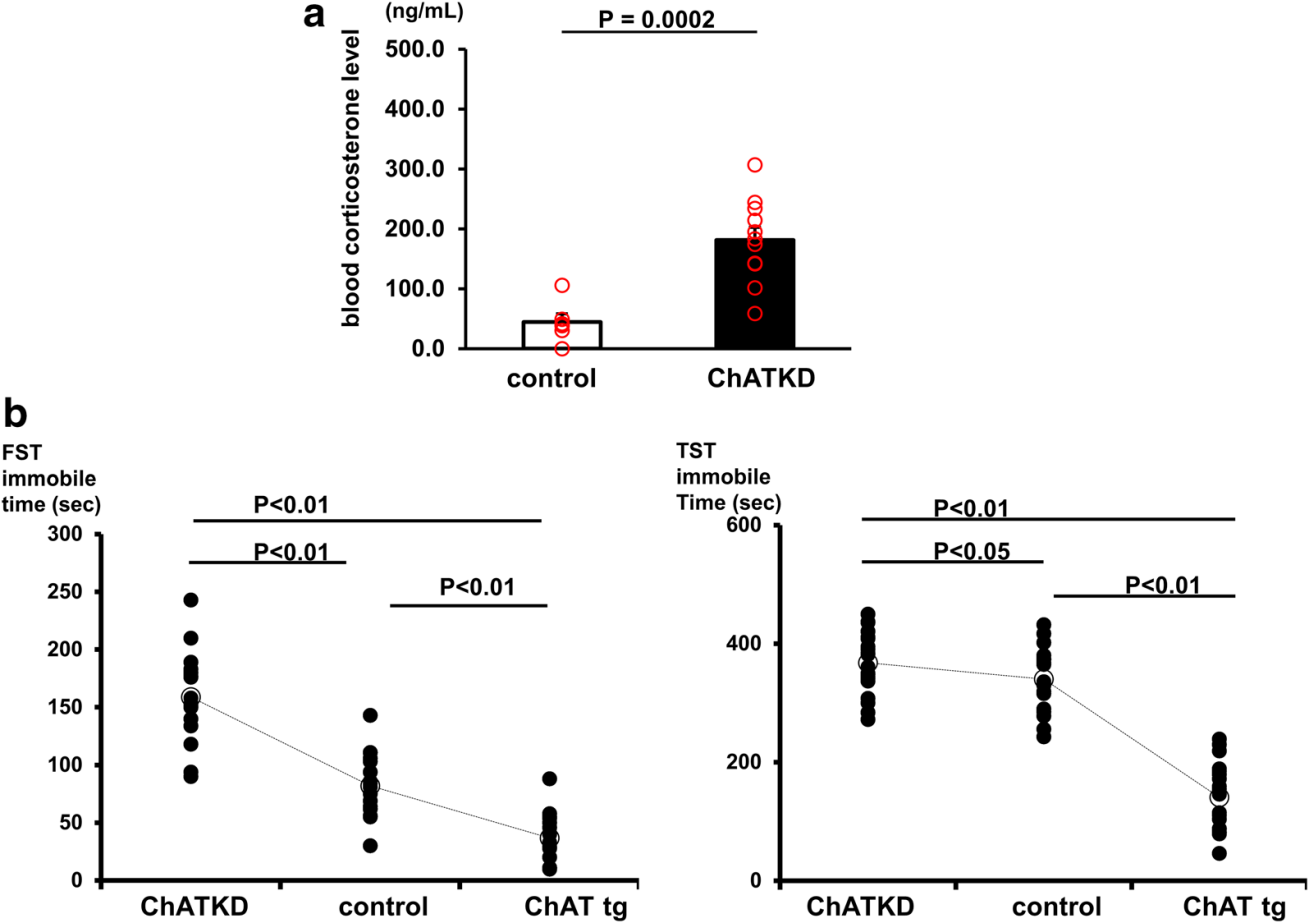
ChATKD hearts are more susceptible to stress and depression. **a** Blood corticosterone levels measured in the morning are higher in ChATKD hearts than in control hearts (*P* = 0.0002). **b** Both FST and TST tests reveal that ChATKD mice show more depression-like symptoms than control mice (*P* < 0.01 and *P* < 0.05, respectively); in contrast, the ChAT tg mice are more resistant to a depression-like episode than control mice (*P* < 0.01)

The NO levels were lower and the vagus nerve activity was more decreased in the ChATKD heart suffering more cardiac dysfunction. Therefore, it was speculated that the ChATKD mice could suffer from depression-like symptoms. We measured blood corticosterone levels in both the ChATKD and control mice to draw a comparison (Fig. 5a). Basal blood corticosterone levels were significantly elevated in the ChATKD mice than in the control mice (181.5 ± 20.9 ng/mL vs. 44.3 ± 14.1 ng/mL, *t* = 5.428, *P* = 0.0002, *n* = 6 − 11) (Fig. 5a). As previously reported, the ChAT tg mice showed less blood corticosterone levels 1 h following restraint stress than control mice [14]. Therefore, the ChATKD and ChAT tg mice possessed opposite responses, suggesting that the ChATKD mice appear to be more depressive than the control mice.

### ChATKD mice show characteristic phenotypes, such as aggravated depression-like symptoms

To examine whether the ChATKD mice showed depression-like symptoms, they were subjected to FST and TST, which are known representative tests. The immobility time in both the tests was significantly longer in the ChATKD mice than in the control mice [FST: 159 ± 9 s vs. 82 ± 7 s, F(2, 48) = 71.74, *P* < 0.01; TST: 367 ± 9 s vs. 332 ± 10 s, F(2, 77) = 107.7, *P* < 0.05] (Fig. 5b), suggesting that the ChATKD mice showed depression-like symptoms. In contrast, the ChAT tg mice clearly showed a significant decrease in immobility in both the tests (FST: 37 ± 5 s vs. control, *P* < 0.01; TST: 142 ± 11 s vs. control, *P* < 0.01).

#### The ChATKD mice show an aggression phenotype

**Fig. 6:**
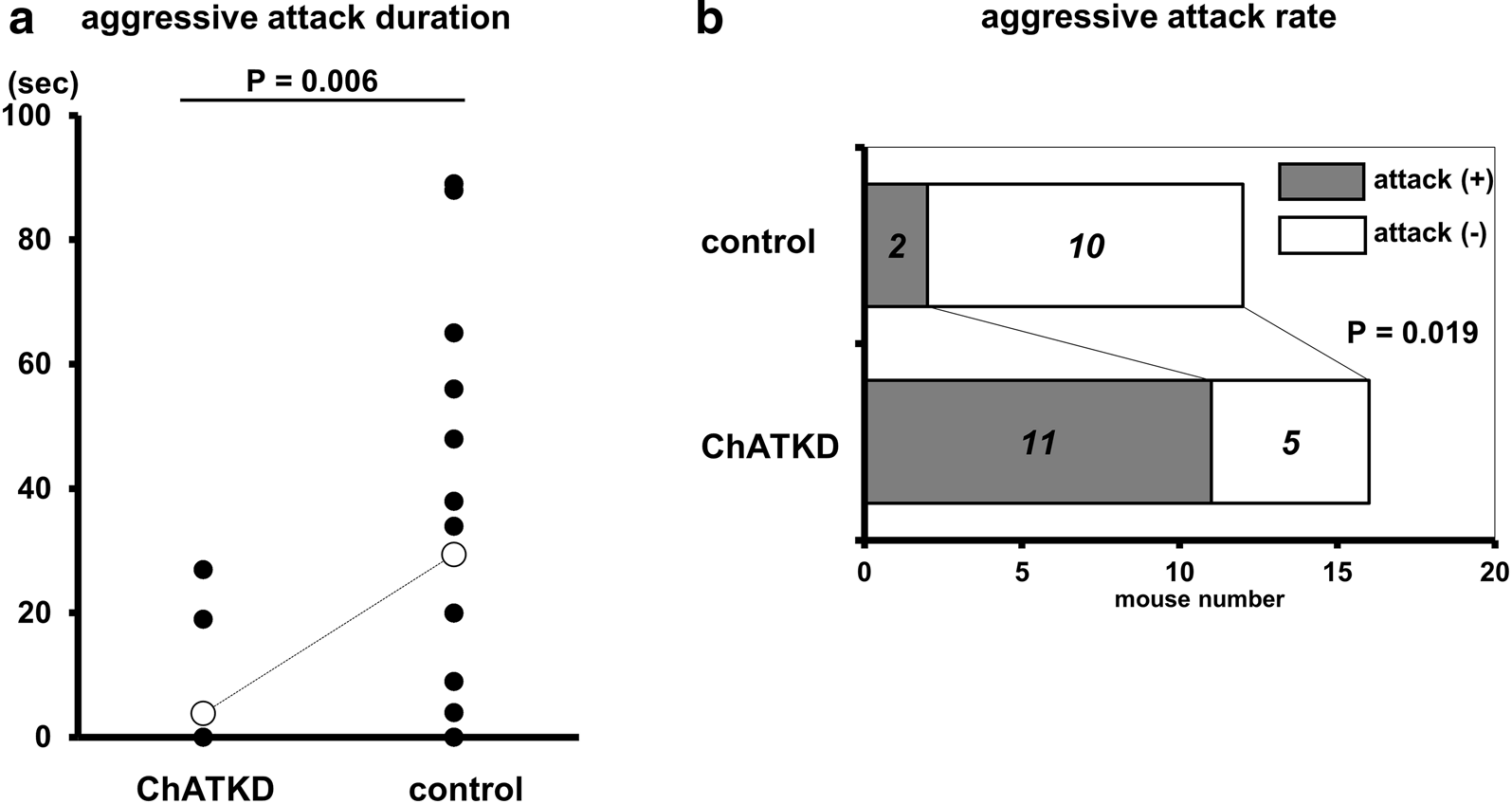
ChATKD mice possess more aggressive characters. **a** The ChATKD mice continue to attack for longer durations against the intruder than control mice (*P* = 0.006). **b** The aggressive attacking rate is also higher in ChATKD mice than in control mice (*P* = 0.019)

The other characteristic phenotype was identified in ChATKD mice, i.e., aggression. Aggressive attacking durations and patterns, recorded by a digital camera, showed that ChATKD mice more frequently and aggressively chased and attacked an intruder for a prolonged duration within the limited observation time (29 ± 8 s vs. 4 ± 3 s, *t* = 3.077, *P* = 0.006, *n* = 12–16) (Fig. 6a). This aggression phenotype was also supported by the ChATKD aggressive rate. As shown in Fig. 6b, the attack rate was significantly higher in the ChATKD mice than in the control mice (ChATKD vs. control, 68.8% vs. 16.7%, *P* = 0.019, *n* = 12–16).

## Discussion

During the start of our study, because of a technical limitation in developing knockout mice at that time, we selected another way to develop transgenic mice possessing commercially available and Invitrogen™-validated murine ChAT-specific siRNAs [7], which were specifically overexpressed in the heart. Furthermore, our vector possessed a special advantage, i.e., three different ChAT-specific siRNAs targeting some common 3′-UTR sites were driven by the heart-specific promoter α-MHC [9]. Therefore, when the vector was restrictively expressed in the heart, three different ChAT siRNAs were simultaneously expressed to efficiently downregulate the cardiac ChAT mRNA expression, leading to ChATKD development. The ChAT mRNA and protein levels were lower in the ChATKD heart than in the control heart, suggesting that ChATKD mice downregulated cardiac NNCCS, and the cardiac ACh synthesis was lower in the ChATKD heart, but it was not completely deleted because cardiac ACh levels of ChATKD hearts were almost a half of control levels. As clarified by this study, even such a reduction level of cardiac ACh caused cardiac and extra-cardiac phenotypes, suggesting that NNCCS is crucial for the heart to sustain a basal ACh level, preventing impediment of cardiac homeostasis.

### The ChATKD mice presenting cardiac dysfunction phenotypes after reaching the adult age

Our previous study clearly demonstrated that the heart possesses NNCCS to constitutively synthesize ACh up to the indispensable level to maintain the cardiac fundamental functions [1, 7, 15], for example, sustaining gap junction functions [7], angiogenesis [10], energy metabolism regulation [1, 7, 9], and protecting the heart from ischemic/hypoxic insults and augmented sympathetic nervous system [6, 7, 9, 15]. Our in vitro study strengthened the above findings, since in the case of attenuated non-neuronal ACh system, the cells suffered from more increased oxygen consumption and ROS exposure leading to apoptosis [1, 7]. However, in contrast to those gene-overexpressing models, there are a few in vivo experiments using downregulated NNCCS models, which include systemic ChAT gene deletion [11] and the cre–loxP system-mediated heart limited VAChT and ChAT gene deletion [6, 12], in which the hearts showed depressed cardiac responses after physiological and pathological stress, clearly suggesting that NNCCS is indispensable for cardia homeostasis.

Initially, the ChATKD mice showed decreased SBP and DBP after 4 months of age as well as decreased HR. The reduction of all parameters including SBP, DBP, and HR suggested that the ChATKD mice may suffer from cardiac dysfunction, as also reported in the previous study that HR is not increased but rather decreased in heart failure [16]. These findings prompted us to rigorously measure ChATKD cardiac function. Intriguingly, the results were compatible with malfunctioned cardiac function because ESP, SV, CO, and EF declined in the ChATKD heart. Supportively, ChATKD hearts expressed upregulation of ANP and BNP mRNAs and showed more fibrotic changes between the muscle fibers, evidenced by a histological analysis. All these results were compatible with a failing heart, and therefore, ChATKD mice were identified to develop cardiac dysfunction.

These results should be more profoundly considered because the NNCCS had never been expected to be such an indispensable system for cardiac homeostasis in vivo until the cardiac dysfunction was found in the ChATKD mice. This may be the different finding from those of the previous studies [6, 12], indicating abnormal cardiac responses after varied stress loading but normal cardiac function in non-stressed conditions. The underlying mechanisms leading to such differences may be due to the different models, however, our findings are partly supported by the previous one [6, 12].

### NNCCS influences glucose energy metabolism in the heart through HIF-1α/Akt/PDK4 and fatty acid metabolism through PGC-1

Our previous studies revealed that one of the various NNCCS functions involved the energy metabolism modulation in the heart, specifically its glucose metabolism [9, 17, 18]. Expression levels of the glucose metabolism-related factors were downregulated, e.g., HIF-1α, pAkt, and GLUT4, in contrast, they were associated with PDK4 upregulation. Our previous study has already identified that ACh post-translationally regulates a HIF-1α level, which is known as a master transcription factor responsible for glucose metabolism [1]. Therefore, reduced HIF-1α levels in hearts may be attributed to decreased cardiac ACh levels in ChATKD mice. Moreover, these results suggest that both glucose uptake and metabolism may be impaired in ChATKD hearts because PDK4 is an inhibitory kinase for PDH, a key enzyme playing as a limiting gate for the TCA cycle. Therefore, increased PDK4 expression suggests the suppression of pyruvate flow into the TCA cycle through the glycolysis pathway [18–21]. As another aspect of dampened glucose metabolism in ChATKD hearts, the mice showed transcriptional and translational upregulation of gluconeogenesis in the liver, in great contrast to downregulated gluconeogenesis in the ChAT tg liver, compared with the control mice liver. These findings of the oppositely regulated hepatic gluconeogenesis between ChATKD mice and ChAT tg mice may suggest the significance of NNCCS on efficient glucose utilization not limited to the heart, and furthermore, NNCCS may influence its effect on the whole body probably through the vagus nerve because our previous studies with ChAT tg mice revealed the heart-derived activation of the central parasympathetic nervous system [14].

In contrast, expression of a fatty acid oxidation-regulating factor, PGC-1, was significantly depressed in the ChATKD hearts. The heart predominantly utilizes fatty acid oxidation (FAO) as an energy substrate under a normoxic condition to produce more ATP, and utilizes glycolysis to complement the required ATP levels. In contrast, in hypoxic conditions FAO is predominantly downregulated and glucose metabolism is reciprocally upregulated for the compensation; however, the compensation may not usually reach to an adequate level [22–24]. Therefore, in addition to the decreased PGC-1-mediated FAO, if the glucose metabolism impairment further occurs, it may consequently cause further energy shortage in the heart, leading to cardiac dysfunction. However, it is important to be addressed that compared to alteration of glucose metabolism-related protein, the impact of PGC-1 alteration was not remarkable despite the significantly depression, again suggesting that NNCCS mainly influences cardiac glucose metabolism. In addition, reduced VEGF expression may cause impaired angiogenesis in the heart [25], resulting in inadequate oxygen supply to the cardiomyocytes. Both impaired cardiac ChATKD features may exaggerate cardiac dysfunction. Taken together, disturbed glucose metabolism as well as impaired angiogenesis may cause and accelerate heart failure in ChATKD mice.

### NNCCS influences NO production responsible for cardioprotection and interaction of ryanodine receptors

Regarding the cardiac dysfunction pathogenesis, the mice was identified to possess a critical phenotype of failure to synthesize adequate NO in the heart, suggesting that NNCCS is indispensable for synthesis and maintenance of NO. NO possesses pleiotropic actions in the heart [26], which is synthesized in endothelial cells and cardiomyocytes through eNOS (NOS3) and nNOS (NOS1), respectively [27–29]. The cardiomyocytes in vitro synthesize NO in response to ACh [30, 31] and therefore, other than the vagus nerve-derived ACh, the NNCCS-derived ACh is also expected to accelerate the NO production from the cardiomyocytes [14, 30, 31]. Moreover, we previously reported the significance of increased cardiac NO in the ChAT tg mice, which produced the beneficial CNS phenotypes including anti-stress because a NOS inhibitor canceled the phenotypes [14]. In contrast, ChATKD hearts possessed an attenuated expression of NOS1, but not NOS3. Additionally, they had the severely impaired NOS1 and RYR interaction in the heart. NOS1 plays a crucial role in regulating cardiac function with RYR through Ca^2+^ mobilization, indicating that NOS1 suppresses Ca^2+^ leak from the sarcoplasmic reticulum to the cytosol [32]. Therefore, our result suggests that the impaired NOS1–RYR interaction and the decreased NOS1 expression may also play the pathogenesis roles in the failing heart of ChAT KD mice. In other words, NNCCS influences cardiac NO synthesis and cardiac functions. This is another new finding of the current study in addition to its important roles in cardiac glucose metabolism.

### The CNS phenotypes of ChATKD mice influenced by impaired NO synthesis in the heart

Previously, we reported that ChAT tg mice, as referred to as a murine augmented NNCCS model, possessed not only anti-ischemia/hypoxia potentials in the heart but also anti-stress, anti-depressive, and anti-convulsion actions in the CNS, and these phenotypes are tightly mediated by the augmented vagus nerve activity and cardiac NO levels, and consequently, a NOS inhibitor reversed the phenotypes [14]. These ChAT tg phenotypes suggest that augmentation of NNCCS influences both the heart and the extra-cardiac organ, CNS, function via the vagus nerve. The vagus nerve or cholinergic activity has been reported to be critical for sustaining cardiac homeostasis because heart failure or cardiac dysfunction depresses its activity [33, 34]. Recent studies reported that donepezil, an anti-Alzheimer’s disease drug, intriguingly suppressed progression of cardiac dysfunction in patients with Alzheimer’s disease and increased a survival rate from cardiovascular diseases [35, 36]. Although those epidemiologic studies did not specifically mention about the mechanisms for donepezil, they speculated a recovery of the cholinergic system and NNCCS augmentation by donepezil [36]. Based on those reports, our results of reduced vagus nerve activity in ChATKD mice may be related to impaired cardiac NO production.

As evidenced by the current study, the attenuated NO synthesis in the ChATKD heart via downregulated NNCCS may also attribute to the susceptibility to stress and aggression of ChATKD mice because previous studies revealed that nNOS is indispensable for enhancing cardiac vagal function [37] and responsible for regulating aggression phonotypes [38–40]. Being compatible, the ChATKD mice showed an elevated blood corticosterone level, in great contrast to ChAT tg mice with an attenuated peak of corticosterone in restraint stress, suggesting that the ChATKD mice were more susceptible to stress.

Moreover, in both FST and TST, immobile durations in ChATKD mice were longer than that in control mice; however, those in ChAT tg mice were reciprocally shorter compared with in control mice, suggesting that ChATKD mice were in more depressive-like mood, although the presence of failing heart conditions in the mice may further influence the susceptibility of depressive-like phenotype of ChATKD mice.

Additionally, ChATKD mice significantly often attacked an intruder by relentlessly chasing it and biting the back. These findings of ChATKD mice were in great contrast to the control mice and ChAT tg. These findings suggest that downregulation of the NNCCS leading to decreased ACh in the heart deteriorates a stressful condition and aggressive character. As already mentioned, aggression was often observed in nNOS deleted mice [39, 40], and therefore, it may be reasonable for ChATKD mice to possess such a specific phenotype. Taken together, this study demonstrates ChATKD mice possessed evident cardiac and extra-cardiac phenotypes, which were influenced by attenuated cardiac NO signals and decreased vagus nerve activity.

With this regard of ChATKD, a few studies reported the phenotypes of ChAT knockout mice, in which one reported fatal neonate pups using conventional knockout system, but evident phenotypes of cardiac dysfunction only when loaded by stress using the cardiac conditional knockout system [6, 11, 12]. In contrast, our mice cardiac phenotypes became evident gradually and slowly several months after they reached the adult age. Therefore, such an extremely delayed appearance may cause failure to be observed. Furthermore, our study revealed novel phenotypes of the CNS, the extra-cardiac phenotypes, in this model, consolidating that the NNCCS influences not only the heart but also the CNS, probably through the vagus nerve, as shown by our previous study [14].

As a limitation of this study, we could not completely exclude the possibility that even the commercially available and validated specific ChAT siRNA sequence can downregulate the other mRNAs. However, even with the conventional gene knockout technology there may be some possibility to sequentially induce other gene expression modulation following the interested gene deletion. Therefore, this study demonstrates a possibility of the NNCCS having an important role in the regulation of cardiac and CNS functions. Further studies will be expected to profoundly investigate mechanisms in near future.

## Conclusions

This study indicates that the NNCCS is indispensable for constitutive NO production in the heart. Furthermore, by sustaining cardiac ACh and NO within some optimized levels, NNCCS plays a crucial role in protecting the heart by maintaining its physiological functions as well as inducing the CNS anti-stress machinery to keep its condition within a stationary state.

## Data Availability

The datasets during and/or analyzed during the current study available from the corresponding author on reasonable request.

## Abbreviations

NNCCS: The non-neuronal cardiac cholinergic system
NNA: Non-neuronal ACh
ACh: Acetylcholine
ChAT: Choline acetyltransferase
MHC: Myosin heavy chain
HIF: Hypoxia-inducible factor
GLUT4: Glucose transporter 4
TCA: Tricarboxylic cycle
ROS: Reactive oxygen species
NO: Nitric oxide
VAChT: Vesicular ACh transporter
UTR: Untranslated region
WT: Wild type
HPLC: High-performance liquid chromatography
ANP: Atrial natriuretic peptide
BNP: Brain natriuretic peptide
PEPCK: Phosphoenolpyruvate carboxykinase
G6Pace: Glucose-6-phosphatase
PVDF: Polyvinylidene difluoride
PDK: Pyruvate dehydrogenase kinase
PDH: Pyruvate dehydrogenase
PGC-1: Peroxisome proliferator-activated receptor γ coactivator
Sirt: Sirtuin
GFP: Green fluorescent protein
PV: Pressure–volume
HR: Heart rate
ESP: End-systolic pressure
EDP: End-diastolic pressure
ESV: End-systolic volume
EDV: End-diastolic volume
SV: Stroke volume
CO: Cardiac output
EF: Ejection fraction
VEGF: Vascular endothelial growth factor
DAB: Diaminobenzidine
PFA: Paraformaldehyde
NOS: Nitric oxide synthase
RYR: Ryanodine receptor
DAPI: 4′,6-Diamidino-2-phenylindole
SERCA: Smooth endoplasmic reticulum Ca^2^^+^ ATPase
SOD: Superoxide dismutase
MDA: Malondialdehyde
TST: Tail suspension test
FST: Forced swim test
ANOVA: Analysis of variance
FAO: Fatty acid oxidation
CNS: Central nervous system
